# The Interplay Between Antioxidant and Chaperone Functions of α-Crystallin

**DOI:** 10.3390/cells15100937

**Published:** 2026-05-20

**Authors:** Krishna Sharma, Puttur Santhoshkumar, Tenzin Tender

**Affiliations:** 1 Department of Ophthalmology, School of Medicine, University of Missouri-Columbia, Columbia, MO 65212, USA; ttz6p@health.missouri.edu; 2 Department of Biochemistry, University of Missouri-Columbia, Columbia, MO 65211, USA

**Keywords:** crystallin, oxidation, chaperone, cataract, lens

## Abstract

**Highlights:**

**What are the main findings?**

- α-Crystallin functions as both a molecular chaperone and an intrinsic antioxidant that protects lens proteins from oxidative stress and aggregation.
- Oxidative modifications and aging alter α-crystallin structure, impairing its chaperone activity, redox regulation, and cytoprotective functions.

**What are the implications of the main findings?**

- Preservation of α-crystallin antioxidant and chaperone functions may be critical for maintaining lens transparency and preventing cataract formation.
- Targeting redox regulation and proteostasis pathways involving α-crystallin may provide novel therapeutic strategies for age-related ocular diseases.

**Abstract:**

α-Crystallin, the predominant protein of the eye lens, possesses molecular chaperone activity and antioxidative properties, both of which are essential for maintaining lens transparency. Its chaperone function prevents the formation of light-scattering protein aggregates, while its antioxidative activity mitigates oxidative stress through both direct and indirect mechanisms. However, with aging, α-crystallin undergoes cumulative post-translational modifications and oxidative damage, leading to protein crosslinking and a decline in chaperone efficacy. Notably, α-crystallin exhibits free radical-scavenging activity comparable to that of serum albumin, a well-characterized antioxidant protein. In addition, its ability to bind redox-active metal ions and convert them into redox-inactive forms significantly reduces reactive oxygen species (ROS) generation in vivo. α-Crystallin also interacts with key proteins and signaling pathways involved in oxidative stress responses, further enhancing its multifunctional protective role. This review summarizes current evidence on the antioxidative properties of α-crystallin and their relationship to its chaperone function, highlighting its importance in lens homeostasis and age-related cataract formation.

## 1. Introduction

Lens α-crystallin is a heterooligomer composed of two subunits, αA and αB, which together account for ~40% of lens proteins [1]. Due to its high stability and short-range order, α-crystallin is considered the primary protein responsible for maintaining lens transparency. In addition to its structural role, α-crystallin is a multifunctional protein with stress-resistance properties, the ability to modulate the redox state of lens proteins, and antiapoptotic activity, as demonstrated in several studies [2,3,4]. It belongs to the small heat shock protein (sHSP) family and exhibits chaperone-like activity [5]. Within the lens, α-crystallin plays a critical role in preserving protein solubility by preventing the aggregation of structurally compromised proteins. Such damage arises from normal aging processes, including oxidative stress, deamidation, truncation, and non-enzymatic glycation, all of which contribute to cataract formation [6]. The essential role of α-crystallin in maintaining lens transparency is further supported by knock-in and knockout studies [7,8]. Therefore, preserving the structural integrity and functional capacity of α-crystallin throughout life is crucial for sustaining lens homeostasis and preventing age-related opacification.

Previous studies have demonstrated that α-crystallin mitigates oxidative stress induced by chemicals such as sodium iodate (NaIO_3_) and hydrogen peroxide (H_2_O_2_). Additionally, binding a divalent metal ion to α-crystallin significantly reduces its redox activity, thereby limiting its capacity to generate ROS [9]. Earlier studies have found that α-crystallin exhibits antioxidant properties [10]. The ability of α-crystallin to protect thiol groups in proteins from oxidation [11], suppress UV-radiation-induced aggregation [12], elevate intracellular glutathione levels [13], and protect cellular lipids [14] was interpreted as supportive of the antioxidant property of the protein. By direct measurement of free radical scavenging properties of bovine α-crystallin, Manzanares reported that the lens protein has antioxidant properties comparable to those of bovine serum albumin [10].

This review summarizes the antioxidative properties of human lens α-crystallin and its interplay with chaperone activity in the context of protein oxidation. Given that chaperone activity is essential for maintaining lens transparency, age-related oxidative insults that accumulate in α-crystallin play a critical role in determining lens homeostasis and clarity. We highlight evidence supporting the antioxidant function of α-crystallin, including its ability to suppress the redox activity of divalent metal ions in vivo. Additionally, we review the capacity of α-crystallin to mitigate the effects of low-molecular-weight oxidants, such as H_2_O_2_ and hypochlorous acid (HOCl), as well as other toxic chemicals, including NaIO_3_ and paraquat.

## 2. Antioxidative Properties of α-Crystallins

The antioxidative and free radical scavenging properties of α-crystallin were extensively investigated by Manzanares et al. [10] using purified bovine lens α-crystallin in multiple in vitro antioxidant assays. These studies demonstrated that α-crystallin effectively suppresses spontaneous lipid peroxidation in brain homogenates, scavenges 2,2′-azinobis(3-ethylbenzothiazoline-6-sulfonic acid) (ABTS)-derived radical cations, traps peroxyl radicals, and neutralizes hypochlorous acid (HOCl), a potent oxidizing agent generated during inflammatory responses. In several assays, the antioxidant activity of α-crystallin was comparable to or greater than that of bovine serum albumin (BSA), a well-characterized antioxidant protein [12,13].

Using the oxygen radical antioxidant capacity (ORAC) assay, the authors showed that 1 mg of α-crystallin exhibited antioxidant activity equivalent to approximately 5 µM Trolox, closely resembling the activity of BSA. In ABTS radical bleaching assays, α-crystallin trapped slightly more radicals and reacted more rapidly than BSA, indicating efficient free-radical scavenging. α-Crystallin also strongly protected c-phycocyanin from HOCl-mediated oxidation, suggesting rapid interaction with and neutralization of reactive oxidants. Collectively, these findings established that α-crystallin is an intrinsic antioxidant protein that directly interacts with reactive oxygen species (ROS) and free radicals.

Additional evidence supporting the antioxidative role of α-crystallin was provided by Masilamoni et al., who demonstrated that exogenously administered α-crystallin suppresses oxidative stress and lipid peroxidation in inflammation-induced mice [14]. α-Crystallin treatment significantly reduced intracellular ROS levels in lymphocytes, hepatocytes, and astrocytes, restored antioxidant enzyme activities, including superoxide dismutase, catalase, and glutathione peroxidase, and decreased lipid peroxidation in plasma, liver, and brain tissues. The protein also inhibited autooxidation-induced lipid peroxidation in cerebral cortex homogenates and preserved membrane-associated ATPase activities under oxidative stress conditions. These findings support the concept that α-crystallin functions not only as a molecular chaperone but also as a multifunctional antioxidant and cytoprotective protein.

Despite these observations, several important questions remain unresolved. In their study, Manzanares et al. [10] did not separate the αA- and αB-crystallin subunits; therefore, their relative contributions to antioxidant activity remain unclear. Furthermore, α-crystallin is an exceptionally long-lived protein that undergoes extensive age-related post-translational modifications, including truncation, deamidation, oxidation, and glycation [15,16,17]. Although these modifications are known to alter protein structure and chaperone function, their effects on α-crystallin’s antioxidative properties remain poorly understood. Further, it is yet to be determined whether β- and γ-crystallins, also present in lenses, exhibit antioxidant activity and whether age-related modifications affect their antioxidant properties (if any).

## 3. Antioxidative Properties of α-Crystallin Mitigate the Effects of Oxidizing Agents

Multiple studies have demonstrated that α-crystallin protects proteins and cells against oxidative injury induced by a wide range of oxidizing agents, including H_2_O_2_, HOCl, NaIO_3_, selenium compounds, tert-butyl hydroperoxide (tBH), and UV irradiation. Oxidative stress generated by these agents results in ROS production, protein unfolding, lipid peroxidation, mitochondrial dysfunction, and apoptosis [18,19,20,21,22,23,24]. The antioxidative properties of α-crystallin, including those of its αA- and αB-subunits and derived mini-chaperone peptides, have been evaluated using purified proteins, cultured cells, and in vivo animal models exposed to oxidative stress conditions.

Experimental studies have shown that α-crystallins and their derived mini-chaperones protect oxidant-sensitive proteins from aggregation, preserve membrane integrity, inhibit lipid peroxidation, and reduce oxidative cell death [25,26,27]. These protective effects have been demonstrated in cultured lens epithelial cells, retinal pigment epithelial (RPE) cells, *Drosophila*, *C. elegans*, and mouse models. Additional studies have shown that α-crystallin suppresses UV-induced oxidation and aggregation of β- and γ-crystallins and protects membrane lipids from oxidative degradation [28,29]. Collectively, these observations indicate that α-crystallins possess intrinsic antioxidative and cytoprotective properties that extend beyond their classical chaperone activity.

Part of this protective effect may stem from α-crystallin’s chaperone function, specifically, its ability to inhibit protein aggregation under oxidative stress, such as UV- or H_2_O_2_-induced unfolding. However, direct evidence for radical scavenging by α-crystallin has also been documented. Manzanares et al. [10] demonstrated that purified bovine lens α-crystallin can directly interact with free radicals, similar to serum albumin, a well-known antioxidant protein. Their work showed that α-crystallin not only reacts with HOCl and other radicals but also suppresses the oxidative burst of polymorphonuclear leukocytes and inhibits autoxidation of brain extracts. The radical-scavenging ability was quantitatively assessed using the ABTS radical bleaching method [30]. Under similar experimental conditions, α-crystallin trapped 0.053 mM of ABTS radicals per gram of protein, surpassing the 0.045 mM/g radical-scavenging capacity of bovine serum albumin, highlighting α-crystallin’s superior antioxidative potential. Furthermore, α-crystallin effectively neutralizes peroxy radicals, and at physiological lens concentrations (~100 mg/mL), its antioxidant capacity has been estimated to be equivalent to ~0.5 M Trolox [10].

HOCl, a potent oxidizing agent commonly used as an antibacterial compound, is also produced endogenously by activated white blood cells. HOCl is known to modify protein side chains, particularly those of methionine and cysteine residues, and to induce protein crosslinking and aggregation. Kantorow et al. [18] investigated whether αB-crystallin could protect lens epithelial cells and retinal pigment epithelial (RPE) cells from oxidative stress induced by H_2_O_2_ or HOCl. Their study demonstrated that overexpression of αB-crystallin in these cells preserved mitochondrial membrane potential and protected mitochondrial function under oxidative challenge. Notably, αB-crystallin was found to translocate to the mitochondria in response to oxidative stress, where it directly interacted with cytochrome c and protected its Met80 residue from oxidation to Met80 sulfoxide. Furthermore, αB-crystallin was shown to inhibit the acquisition of peroxidase activity by cytochrome c, which typically occurs upon oxidation of Met80. These functions are categorized as indirect antioxidative mechanisms mediated by αB-crystallin.

Sodium iodate is another oxidative agent widely used to evaluate the antioxidative efficacy of biomolecules. It serves as an inducer of retinal degeneration in cell culture and animal models of diseases such as dry age-related macular degeneration (AMD) [19]. Exposure to NaIO_3_, either in vitro or through intravitreal injection, leads to RPE cell death via multiple pathways, including apoptosis, ferroptosis, necroptosis, and pyroptosis [19]. Previous studies have established the antiapoptotic properties of both αA- and αB-crystallins. Mini-chaperone peptides derived from the functional domains of these crystallins have also been shown to recapitulate the protective functions of the full-length proteins [31,32].

**Figure 1:**
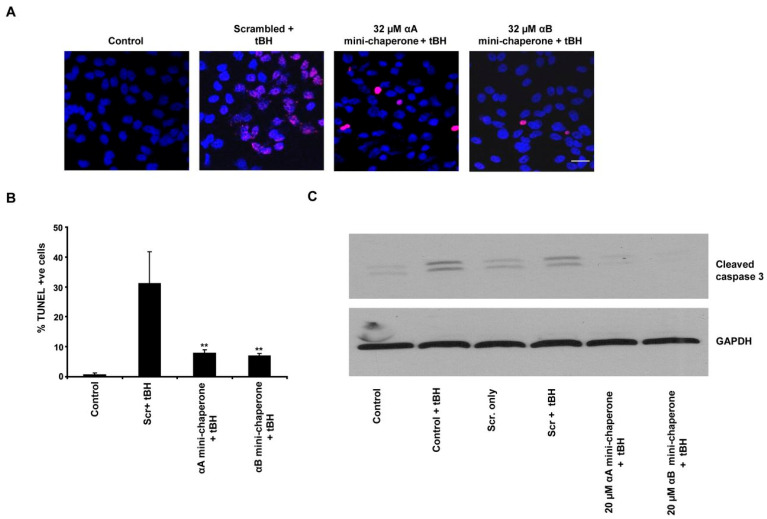
Suppression of oxidant-induced cell death by αA- or αB-crystallin-derived mini-chaperone peptides. (**A**) hfRPE cells were treated with 200 μM tBH or 200 μM tBH plus 32 μM of either αB-crystallin-derived or αA-crystalline-derived mini-chaperone peptide for 4 h. Apoptosis was assessed by TUNEL staining. Confocal images of TUNEL-positive cells (red) and nuclei (blue) with and without cotreatment with αA, αB-crystallin-derived peptides are shown. (**B**) Quantification of percent dead cells by TUNEL assay. Apoptosis was significantly higher in cells cotreated with tBH and scrambled peptide when compared with cells cotreated with either αA- or αB-crystallin-derived mini-chaperone peptide and tBH. Asterisks indicate *p* < 0.01 versus scrambled crystallin-derived peptides treated with tBH. (**C**) Exogenously added α-crystallin mini-chaperones protect hfRPE cells from tBH-induced oxidative stress by inhibiting activation of caspase-3. hfRPE cells were treated with 200 μM tBH either alone or in the presence of 20 μM αA- or αB-crystallin mini-chaperone in serum-free medium for 4 h. Caspase-3 activation was prominent in control cells and in cells cotreated with scrambled peptide and 200 μM tBH. Scr represents scrambled α-crystallin mini-peptide, and αA- and αB- represent αA-crystallin-derived mini-chaperone peptide and αB-crystallin-derived mini-chaperone peptide, respectively (reproduced from [20]).

In a notable study, Sreekumar et al. [20] evaluated whether such mini-chaperone peptides could confer protection against oxidative stress in cultured human fetal RPE (hfRPE) cells. Their results demonstrated that cotreatment with the peptides and tBH significantly reduced oxidative cell death (Figure 1). In contrast, scrambled control peptides failed to provide protection, confirming the sequence-specific action of the chaperone peptides. Western blot analysis further revealed suppression of caspase-3 activation (panel C in Figure 1), supporting the antiapoptotic effect. Similar protective outcomes were observed when hfRPE cells were exposed to H_2_O_2_ and simultaneously treated with nanoparticles encapsulating the mini-chaperones. These findings underscore the antioxidative and cytoprotective properties of chaperone peptides derived from α-crystallins.

In a separate study, a peptide corresponding to the chaperone site of αA-crystallin (αA-mini-chaperone) was shown to protect the mouse retina when administered intravitreally before NaIO_3_ injection [21]. The αA-mini-chaperone significantly reduced NaIO_3_-induced apoptosis and autophagy in RPE cells. Furthermore, it attenuated the expression of CHOP and XBP1, key markers of endoplasmic reticulum (ER) stress, in the treated retinas. In a related study, robust expression of αB-crystallin in the retina was found to suppress NaIO_3_-induced ROS generation [22], suggesting that increasing endogenous α-crystallin levels can protect against oxidative stress. Complementary evidence comes from studies involving α-crystallin-deficient mice, which showed increased susceptibility to oxidative injury following exposure to NaIO_3_ or H_2_O_2_. Supplementation with exogenous αA- or αB-crystallin in these models resulted in protective effects, confirming the antioxidative function of these proteins [22,23,24].

Sodium selenite-induced cataract in young rats is a well-established model of oxidative stress that mimics certain features of human senile cataract, including damage to sulfhydryl groups, protein aggregation, and lens opacification [33]. In this model, intraperitoneal administration of mini-chaperones derived from αA- and αB-crystallin significantly suppressed cataract formation by reducing apoptosis in lens epithelial cells, limiting protein insolubilization, and mitigating oxidative stress [26]. More recently, systemic administration of αA-mini-chaperone peptides has been shown to delay the onset of diabetic cataract in rats by attenuating oxidative and ER stress, as well as the hyperglycemia-induced apoptosis [34]. Further extending these findings, Rao and colleagues demonstrated that recombinant human αB-crystallin, when exogenously administered, could enter mouse neural stem progenitor cells (mNSPCs) and protect them against paraquat-induced oxidative stress [35]. In *Drosophila* models, the expression of αB-crystallin in neurons and the fat body also conferred protection against paraquat toxicity, reinforcing its antioxidative role at the organismal level [25].

Collectively, these studies underscore that the antioxidative properties are inherent to both αA- and αB-crystallins and their derived mini-chaperones. Whether expressed constitutively or introduced exogenously, these proteins exhibit potent protective effects against oxidative stress in various cellular and organismal models.

## 4. Metal Ion Binding to α-Crystallin and Implications for Oxidative Stress

Copper is present in the lens tissue at concentrations ranging from 3 to 10 μM, and the majority of this copper is tightly bound to lens proteins [9,36]. Ortwerth and James [9] reported that this tight binding of Cu^2+^ ions reduces the generation of ROS and minimizes the oxidation of ascorbic acid, thereby decreasing oxidative stress in vivo. Further, a study by Rao and colleagues demonstrated that both αA- and αB-crystallins contribute to redox silencing of bound metal ions, effectively rendering them redox-inactive [37]. Several studies have also shown that α-crystallin undergoes modulation of its chaperone activity upon interaction with Cu^2+^ and other divalent metal ions, such as Zn^2+^ and Ni^2+^ [38,39]. It has been estimated that between one to six Zn^2+^ ions bind to each subunit of α-crystallin with an average stoichiometry of little over three metal ions per subunit [40]. The wide range of Zn^2+^ binding to α-crystallin subunits is likely due to the weak interaction between the metal ion and the protein, as evaluated with a relatively high concentration of zinc. It is also possible that some of the Zn^2+^ might be sequestering into the crystallin oligomers cavity. Collectively, the metal ion interactions with α-crystallin affect its role in maintaining lens transparency, particularly in aging lenses, where metal ion accumulation is known to increase.

**Figure 2:**
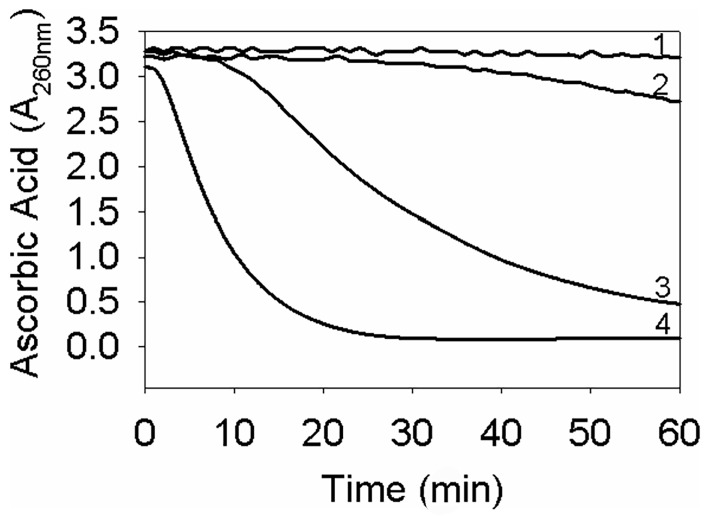
Cu^2+^-induced oxidation of ascorbic acid in the presence of αAΔ70–77 and wild-type αA-crystallin. Ascorbic acid (500 μM) was used in assays. (1) ascorbic acid; (2) + 5 μM Cu^2+^ + 100 μg αAWt; (3) + 5 μM Cu^2+^ + 100 μg αAΔ70–77 and (4) + 5 μM Cu^2+^. The results show that the αA-crystallin lacking metal ion binding site and chaperone site sequences show decreased protection of ascorbic acid from Cu^2+^ (reproduced from [41]).

Our previous work has shown that mini-αA-crystallin peptides, which retain chaperone activity, also bind Cu^2+^ and inhibit copper-induced oxidation of ascorbic acid, much like native α-crystallin and its subunits [41]. Specifically, we identified the αA70–88 region, which encompasses the core chaperone site, as a Cu^2+^-binding domain that is sufficient to suppress Cu^2+^-induced ascorbate oxidation. As illustrated in Figure 2, wild-type αA-crystallin significantly suppresses Cu^2+^-mediated oxidation of ascorbic acid, whereas a deletion mutant lacking residues 70–77 (αAΔ70–77) exhibits markedly reduced antioxidant capacity. Although the 70–77 segment does not contain canonical histidine residues typically involved in metal binding, its deletion likely induces structural changes that compromise the protein’s redox-silencing function. Cu^2+^-binding studies of αAΔ70–77 are ongoing to validate this hypothesis.

The above-stated findings indicate that Cu^2+^ ions bound to α-crystallin, particularly within its chaperone domain, are rendered redox-inactive, thereby preventing free radical generation in metal-catalyzed reactions. This mechanism highlights a critical antioxidative function of α-crystallin, linking its metal-binding capacity to its chaperone activity in protecting lens proteins and maintaining lens transparency under oxidative stress. While the Cu^2+^ binding capacity of α-crystallin is interpreted as redox suppression, the precise role of Zn^2+^ binding to α-crystallin is yet to be delineated. Since bound Zn^2+^ is presumed to protect -SH and imidazole groups, it is possible that Zn^2+^ bound to α-crystallins might be protecting them, as in the case of other proteins [42]. Further study is needed to confirm this.

## 5. α-Crystallin Shows Structural Changes Following Oxidation

It is well established that oxidative stress leads to the chemical modification of specific amino acid residues in proteins, resulting in conformational alterations, crosslinking, aggregation, fragmentation, and the eventual loss of structural and biological function. Due to their longevity and metabolic inactivity, lens crystallins are particularly susceptible to age-related oxidative modifications. These cumulative oxidative insults contribute to protein aggregation and precipitation, ultimately leading to lens opacification, or cataract, one of the primary causes of blindness worldwide [6]. Several studies have focused on understanding the effect of oxidation on lens crystallins.

Rajan et al. [43] investigated the structural consequences of in vitro oxidation of αA- and αB-crystallins using a metal-catalyzed oxidation system (FeCl_3_ + H_2_O_2_). Their study demonstrated significant alterations in both secondary and tertiary structures. Specifically, oxidized αA-crystallin showed a 38% reduction in β-sheet content and a 60% increase in random coil structure. In comparison, oxidized αB-crystallin exhibited a 64% decrease in α-helical content, 63% decrease in β-turns, and a 69% increase in random coils. Corresponding changes in near-UV CD spectra indicated disrupted tertiary structure, including the loss of tryptophan signature peaks and positive ellipticity—hallmarks of altered subunit interactions. Met, His, Arg, Lys, and Cys were likely oxidized in the crystallins, as these are the preferred targets of metal-catalyzed oxidation systems. In addition, Rajan et al. [43] observed that the oxidized αA and αB-crystallin preparations showed degradation and diminished chaperone activity.

More recently, Ghahramani et al. [44] assessed the effects of partial and extensive oxidation of αB-crystallin induced by peroxynitrite or the FeCl_2_ + H_2_O_2_ system. While partial oxidation led to moderate structural changes, extensive oxidation resulted in pronounced protein breakdown, loss of both secondary and tertiary structures, and increased crosslinking. Both mild and extensive oxidation were associated with decreased surface hydrophobicity and diminished protein stability. The findings of Ghahramani et al. [44] were consistent with those previously reported by Rajan et al. [43], reinforcing the structural vulnerability of αB-crystallin under oxidative stress.

Fuiji et al. [45] used gamma radiation (7.8 Gy/s from a ^60^Co source) to modify α-crystallin oxidatively. At doses above 100 Gy, α-crystallin lost most of its tertiary structure and exhibited protein crosslinking, as visualized by SDS-PAGE. Methionine oxidation and isomerization/racemization of Asp151 were also observed. Since gamma irradiation produces hydroxyl radicals, these modifications were likely due to radical-mediated oxidative assault.

Kaiser et al. [46] explored structural consequences of oxidative disulfide bond formation in αA-crystallin, which contains two cysteine residues (Cys131 and Cys142) located on β8 and β9 strands. These residues point in opposite directions, and their disulfide bond necessitates partial protein unfolding. Circular dichroism spectroscopy of oxidized αA-crystallin revealed altered tertiary structure, particularly at the N-terminal region, consistent with Rajan et al.’s [43] findings. Electron microscopy showed that oxidized αA-crystallin formed more polydisperse and larger oligomers than the reduced form, with the average oligomer size increasing from ~13.5 nm to ~17.7 nm. Correspondingly, molecular mass increased from ~380 kDa to ~770 kDa. Crosslinking studies confirmed altered subunit interactions, and hydrogen-deuterium exchange revealed greater solvent exposure of the β9 strand, which harbors Cys142. Oxidized αA-crystallin oligomers were also less stable, requiring lower urea concentrations to dissociate.

Collectively, these studies provide compelling evidence that oxidation induces significant conformational and functional changes in α-crystallins, compromising their structural integrity and chaperone function. Such oxidative alterations are central to cataractogenesis and represent a key therapeutic target for delaying or preventing lens opacification.

## 6. Oxidation and Modifications Disrupt α-Crystallin Chaperone Activity and Antiapoptotic Property

During aging of the human lens, α-crystallins accumulate oxidative and post-translational modifications that progressively impair their structure and function. Oxidation of the two sulfhydryl groups in αA-crystallin can occur as early as 4–5 months of age; however, a pronounced increase in intramolecular disulfide bond formation is typically observed after the third decade of life. This age-dependent accumulation of oxidative damage is driven by sustained exposure to ROS and the exceptional longevity of lens proteins, which renders them particularly vulnerable to modification [6,47]. In addition to direct oxidation, age-related processes such as glycation, deamidation, truncation, and increased levels of redox-active metal ions (e.g., Fe^2+^ and Cu^2+^) further exacerbate oxidative stress through Fenton-type reactions. These processes selectively target susceptible amino acid residues. For example, Met1 and Met138, Trp9, and Tyr18 and Tyr34 in αA-crystallin and Met1, Met68, His7, Trp9, Trp60, and Tyr48 in αB-crystallin are particularly prone to oxidative modification during aging or under oxidative stress conditions.

The molecular chaperone activity of αA- and αB-crystallins is essential for maintaining lens transparency by preventing aggregation of partially unfolded proteins [5]. Genetic studies, including knockout and knock-in models, have firmly established that disruption of α-crystallin function leads to protein aggregation, light scattering, and cataract formation [7,8]. Consistent with this, age-related decline in chaperone activity, coupled with the accumulation of aggregated proteins, directly contributes to lens opacification. With this background, several studies have examined the impact of oxidation on α-crystallin chaperone function. Rajan et al. [43] compared native and H_2_O_2_-oxidized recombinant αA- and αB-crystallins using EDTA-induced aggregation of alcohol dehydrogenase and DTT-induced insulin aggregation at 37 °C. Oxidized crystallins showed a 10–16% reduction in chaperone activity. In another study, Cherian and Abraham [48] observed an 80% loss of chaperone function in α-crystallin that was extensively oxidized with H_2_O_2_ + FeCl_2_ in a heat-induced (60 °C) β_L_-crystallin aggregation assay. In another study, Kaiser et al. [46] showed that reduced and oxidized αA-crystallin suppressed heat-induced aggregation of malate dehydrogenase, although oxidized αA-crystallin was significantly less effective. Interestingly, they demonstrated that oxidized αA-crystallin could transfer its intramolecular disulfide bond to destabilized p53, suggesting a redox-regulatory function. The role of α-crystallin in thiol-disulfide exchange and redox regulation has been reviewed recently [49] and discussed by others [50].

The extent of oxidation appears to regulate α-crystallin function differentially. Ghahramani et al. [44] demonstrated that partial oxidation of αB-crystallin (e.g., 1 mM peroxynitrite or 0.05 mM H_2_O_2_) enhances chaperone activity toward several client proteins, including insulin, lysozyme, and γ-crystallin. This enhancement is likely due to mild structural perturbations that increase exposure of hydrophobic surfaces critical for substrate binding. However, this effect is substrate-dependent and less evident with catalase. In contrast, extensive oxidation (e.g., 30 mM peroxynitrite or 1 mM H_2_O_2_) results in a marked loss of chaperone activity, consistent with structural destabilization and protein damage. This biphasic response to oxidative stress is consistent with earlier findings showing that mild perturbations induced by heat or urea can enhance chaperone activity by promoting subunit rearrangement and increasing substrate accessibility [51,52,53]. However, excessive oxidation leads to irreversible structural alterations, including methionine oxidation, disulfide crosslinking, and aggregation, ultimately compromising protein function. Supporting this, Fuji et al. [45] demonstrated that γ-irradiation of α-crystallin at 4000 Gy reduces chaperone activity by approximately 40%, with further losses observed at higher doses due to extensive oxidative damage and crosslinking.

Collectively, these findings demonstrate that oxidative modifications exert a dual effect on α-crystallin function. While mild oxidation may transiently enhance chaperone activity through structural activation, extensive oxidation leads to loss of chaperone and antiapoptotic functions. This progressive decline in functional capacity plays a central role in age-related protein aggregation and cataract formation.

## 7. Reduction of α-Crystallin from Human and Bovine Lenses Partially Restores Chaperone Activity

**Figure 3:**
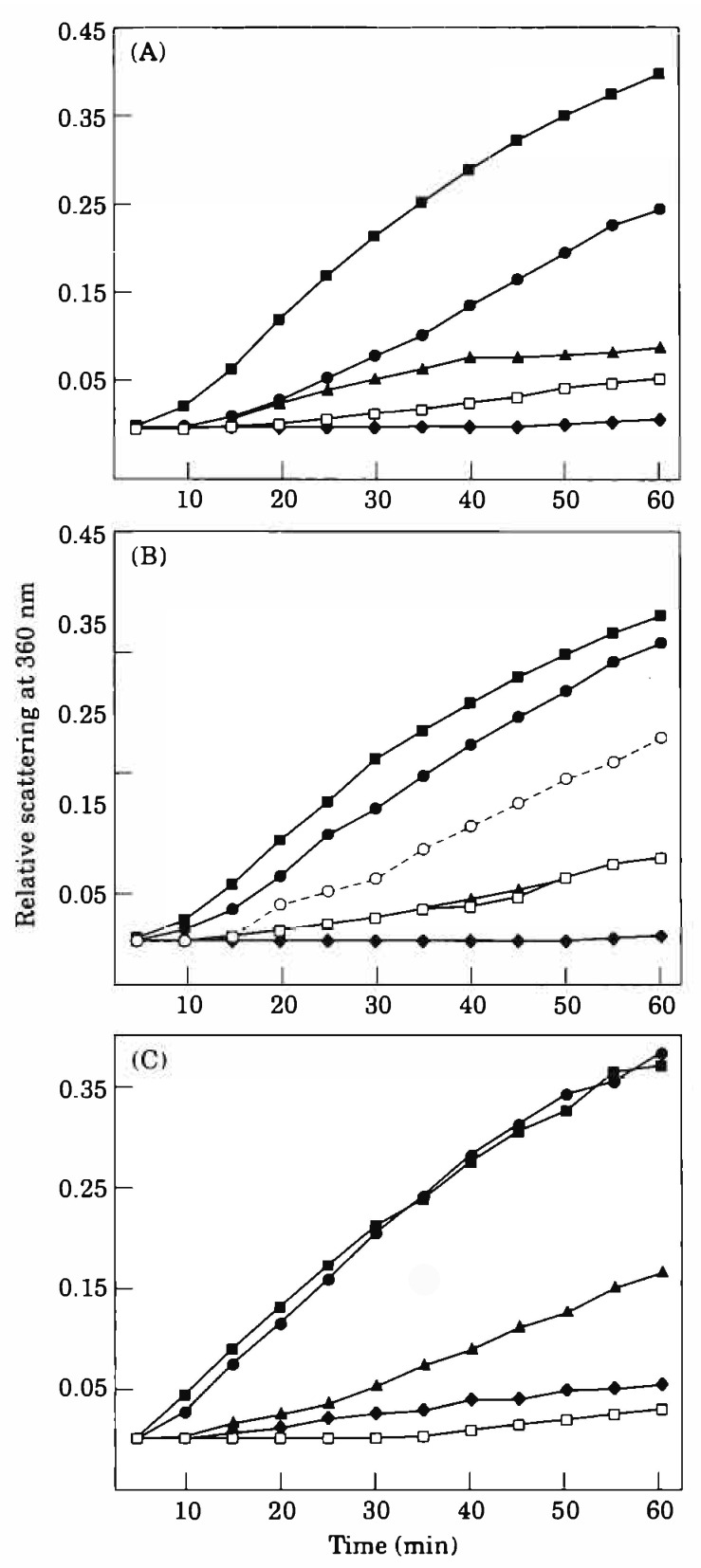
Effect of WISS (water-insoluble supernatant after sonication) and DTT-treated WISS proteins on aggregation of β_L_-crystallin at 56 °C. In each experiment, 190 µg of β_L_-crystallin and varying amounts of WISS in 50 mM sodium phosphate buffer, pH 7.0, were heat-treated, and the aggregation was measured as described in the Methods. To determine the effect of the reduction of disulfides in WISS on chaperone-like activity, bovine and human lens WISS were treated with 5 mM DTT for 2 h before the assay. (**A**) Influence of bovine lens cortical WISS on the thermal aggregation of β_L_-crystallin. (**B**) Influence of bovine lens nuclear WISS on the thermal aggregation of β_L_ crystallin. (**C**) Influence of human lens WISS on the thermal aggregation of β_L_-crystallin. (**A**) ■, β_L_; ●, β_L_ + 14 µg WISS; ▲, β_L_ + 14 µg WISS + DTT; ▢, β_L_ + 28 µg WISS; ♦, β_L_ + 14 µg α. (**B**) ■, β_L_; ●, β_L_ + 14 µg WISS; ○, β_L_ + 28 µg WISS; ▲, β_L_ + 70 µg WISS; ▢, β_L_ + 28 µg WISS + DTT; ♦, β_L_ + 14 µg α. (**C**) ■, β_L_; ●, β_L_ + 16 µg WISS; ▲, β_L_ + 140 µg WISS; ♦, β_L_ + 140 µg WISS + DTT; ▢, β_L_ + 14 µg α. (Reproduced from [54]).

A decline in the chaperone activity of α-crystallin isolated from aged human lenses has been well documented [54]. We demonstrated that α-crystallin present in the water-insoluble (WISS) fraction of both bovine and human lenses exhibits significantly enhanced chaperone activity after treatment with dithiothreitol (DTT), a reducing agent that cleaves disulfide bonds (Figure 3). This observation suggests that oxidation-induced disulfide formation contributes to the reduced chaperone activity observed in aged lens crystallins, particularly within the insoluble protein fraction. However, DTT treatment did not fully restore the chaperone activity to the levels observed in α-crystallin derived from the water-soluble (WSS) protein fraction (Figure 3A–C). This incomplete recovery indicates that additional age-related post-translational modifications, such as truncation, deamidation, and non-enzymatic glycation, also contribute to the functional impairment of α-crystallin in the insoluble fraction. This interpretation is consistent with previous studies demonstrating that such modifications adversely affect protein stability and chaperone function [55,56,57,58,59].

Previous work has also established a direct correlation between the rate of subunit exchange in α-crystallin oligomers and their chaperone activity. Specifically, enhanced subunit exchange is associated with improved chaperone function [60,61]. The increased chaperone activity observed in DTT-treated WISS fractions may thus result from the removal of oxidative constraints that otherwise restrict subunit mobility and exchange within the oligomer.

This interpretation is supported by earlier experiments using a cleavable chemical crosslinker, dimethyl 3,3′-dithiobispropionimidate-2HCl (DTBP), which mimics disulfide bonds. When α-crystallin was crosslinked with DTBP, a marked decrease in chaperone activity was observed in assays using unfolding alcohol dehydrogenase as a substrate. Subsequent treatment with DTT to cleave the crosslinker restored approximately 60% of the original chaperone activity [54]. These results demonstrate that crosslinking, whether chemical or disulfide-mediated, inhibits chaperone function by limiting subunit exchange dynamics.

Taken together, these findings suggest that age-related disulfide crosslinking in αA-crystallin contributes to the decline in chaperone activity in aging lenses. By imposing structural rigidity and limiting subunit exchange, oxidative crosslinking compromises α-crystallin’s protective function, thereby contributing to protein aggregation, loss of lens transparency, and cataract formation.

## 8. Conclusions

α-Crystallin, a member of the sHSP family, plays a crucial role in maintaining lens transparency by functioning as both a molecular chaperone and an antioxidant. The α-crystallin also exhibits notable free radical scavenging activity, with antioxidant properties comparable to those of serum albumin. However, the effects of aging and age-related post-translational modifications on the antioxidative properties of α-crystallins remain to be investigated. Despite the presence of endogenous antioxidant systems, including glutathione and peroxidases, and the intrinsic antioxidative properties of lens crystallins, age-related oxidative damage accumulates over time, primarily due to the long lifespan of these proteins. The chaperone activity of α-crystallin appears particularly sensitive to its redox state, as oxidation alters the oligomeric dynamics essential to its function. The properties of α-crystallin, as summarized in this review, suggest that, beyond its structural and chaperone functions in the lens, its antioxidant capacity may also play a critical role in lens proteostasis as a redox regulator. Future studies aimed at preserving or restoring the antioxidative and chaperone functions of lens crystallins, especially α-crystallin, may offer therapeutic potential for delaying or preventing age-related protein aggregation and the subsequent development of lens opacity and cataract.

## Data Availability

No new data were created or analyzed in this study.

## Abbreviations

The following abbreviations are used in this manuscript:

ABTS	2,2′-azinobis(3-ethylbenzothiazoline-6-sulfonic acid)
AMD	age-related macular degeneration
αA	αA-crystallin
αB	αB-crystallin
α-crystallin	alpha-crystallin
BSA	bovine serum albumin
CD	circular dichroism
Cu^2+^	copper ion
DPPH	1,1-diphenyl-2-picrylhydrazyl
DTBP	dimethyl 3,3′-dithiobispropionimidate-2HCl
DTT	dithiothreitol
ER	endoplasmic reticulum
H_2_O_2_	hydrogen peroxide
HOCl	hypochlorous acid
hfRPE	human fetal retinal pigment epithelial cells
mNSPCs	mouse neural stem/progenitor cells
NaIO_3_	sodium iodate
PBS	phosphate-buffered saline
ROS	reactive oxygen species
RPE	retinal pigment epithelium
sHSP	small heat shock protein
tBH	tert-butyl hydroperoxide
TUNEL	terminal deoxynucleotidyl transferase dUTP nick end labeling
UV	ultraviolet
WISS	water-insoluble supernatant
WSS	water-soluble supernatant
